# Effects of the Pathogenic Mutation A117V and the Protective Mutation H111S on the Folding and Aggregation of PrP106-126: Insights from Replica Exchange Molecular Dynamics Simulations

**DOI:** 10.1371/journal.pone.0125899

**Published:** 2015-05-20

**Authors:** Lulu Ning, Dabo Pan, Yan Zhang, Shaopeng Wang, Huanxiang Liu, Xiaojun Yao

**Affiliations:** 1 State Key Laboratory of Applied Organic Chemistry and Department of Chemistry, Lanzhou University, Lanzhou, China; 2 School of Pharmacy, Lanzhou University, Lanzhou, China; 3 State Key Laboratory of Quality Research in Chinese Medicine, Macau Institute for Applied Research in Medicine and Health, Macau University of Science and Technology, Taipa, Macau, China; 4 School of pharmaceutical technology, Qiandongnan National Polytechnic, Kaili, China; Wake Forest University, UNITED STATES

## Abstract

The fragment 106-126 of prion protein exhibits similar properties to full-length prion. Experiments have shown that the A117V mutation enhances the aggregation of PrP106-126, while the H111S mutation abolishes the assembly. However, the mechanism of the change in the aggregation behavior of PrP106-126 upon the two mutations is not fully understood. In this study, replica exchange molecular dynamics simulations were performed to investigate the conformational ensemble of the WT PrP106-126 and its two mutants A117V and H111S. The obtained results indicate that the three species are all intrinsically disordered but they have distinct morphological differences. The A117V mutant has a higher propensity to form β-hairpin structures than the WT, while the H111S mutant has a higher population of helical structures. Furthermore, the A117V mutation increases the hydrophobic solvent accessible surface areas of PrP106-126 and the H111S mutation reduces the exposure of hydrophobic residues. It can be concluded that the difference in populations of β-hairpin structures and the change of hydrophobic solvent accessible areas may induce the different aggregation behaviors of the A117V and the H111S mutated PrP106-126. Understanding why the two mutations have contrary effects on the aggregation of PrP106-126 is very meaningful for further elucidation of the mechanism underlying aggregation and design of inhibitor against aggregation process.

## Introduction

Prion diseases, such as bovine spongiform encephalopathy, scrapie disease in sheeps, Creuzfeldt-Jacob disease and Gerstmann-Straussler-Scheinker disease in humans, are characterized by accumulations of abnormal forms of prion protein (PrP^Sc^) in neuronal tissue[1–3]. Prion disorders are fatal neurodegenerative diseases that are closely linked to misfolding and aggregation of the cellular prion protein (PrP^C^). Although PrP^C^ and PrP^Sc^ share identical primary structure[4], it has been shown that there are large biophysical differences between them. PrP^C^ has a high content of α-helix, while PrP^Sc^ has a high content of β-sheet[5,6]. Moreover, PrP^C^ is soluble, monomeric and protease K sensitive, while PrP^Sc^ tends to aggregate into insoluble and protease K resistant amyloid fibril [1,2,7]. According to the “protein only hypothesis” [3,8], PrP^Sc^ acts as a template to induce the misfolding of cellular prion protein and is considered to be the causative agent. The mechanism underlying the spontaneous conversion from PrP^C^ to PrP^Sc^ has not been completely elucidated.

A fragment of prion protein, PrP106-126, is highly conserved among various species and displays similar characteristics to the full-length prion protein [9,10]. PrP106-126 is thought to be one of the most critical domains involved in fibril formation and structural transition from cellular prion to its scrapie form. This peptide can induce neuro-pathological alterations observed in prion diseases such as apoptosis of nerve cells, proliferation and hypertrophy of astrocytes [11–13]. It has been also reported that the selective deletion of residues 108–121 or 122–140 along with residues 23–88 can prevent the formation of PrP^Sc^, while only removing residues 23–88 does not inhibit structural conversion, which indicate that PrP106-126 may play an important role in PrP^C^-PrP^Sc^ transition [14]. Furthermore, the peptide tends to aggregate into amyloid fibrils that are resistant to protease [15]. More interestingly, the toxicity of PrP106-126 requires the expression of PrP^C^ [16], which is similar to PrP^Sc^ and further indicates that the toxic mechanism of PrP106-126 may reflect the pathology of PrP^Sc^ [17].

Many efforts have been made to investigate the properties of PrP106-126. The physicochemical nature, aggregating propensity and conformational properties of PrP106-126 have been studied by applying biophysical techniques such as circular dichroism spectrum, nuclear magnetic resonance, ion mobility mass spectrometry, as well as molecular simulations[18–26]. It has been shown that PrP106-126 displays structural diversity and different physical chemical conditions such as solvent composition, ionic strength and pH affect the secondary structure and aggregating propensity of PrP106-126[20,21,27,28]. Mutations of some residues in the peptide also change the conformations and fibrilogenic propensity of PrP106-126. A substitution of alanine by valine at position 117 is related to Gerstmann-Straussler-Scheinker disease [29]. The inclusion of the point mutation has been found to enhance the toxicity of PrP106-126 [30] and increase β-sheet content in the fibrils [31]. It has been suggested that the A117V mutation destabilizes the helix conformation of PrP106-126 significantly [21]. Daidone et al. [32] have proposed that the A117V mutant can change the free energy landscape of PrP109-122 and increase its amyloidogenicity. Contrary to the A117V mutation, the H111S mutation completely abolishes the aggregation of PrP106-126[33]. Despite of the aforementioned studies, there is still much unknown about how mutations such as A117V and H111S affect the folding and aggregation of PrP106-126. The comprehension of impacts of the two mutations on the structure of PrP106-126 will help to understand how different structural elements promote or inhibit aggregation. Understanding the monomer structure is helpful to investigate the mechanism of amyloid formation since the structure preference of monomer may give insight into the amyloid prone states. Due to the tendency of aggregation, the structure of monomeric PrP106-126 in solution is difficult to be resolved experimentally, and little is known about the impact of the above mutations on the structures of the peptides.

In this study, replica exchange molecular dynamics simulations (REMD) were performed to explore the conformational features of monomers of the WT PrP106-126 and its two mutants A117V and H111S. This work can probe the structural features of PrP106-126 and its mutants, which could provide deeper understanding in the aggregation mechanism of PrP106-126 and give further insights into the pathogenesis of prion diseases.

## Materials and Methods

**Fig 1:**
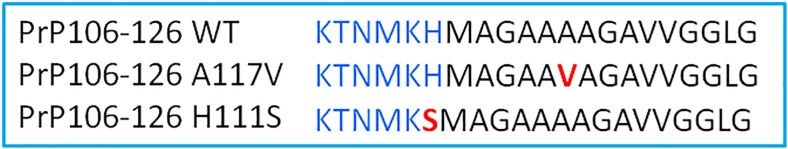
The sequences of WT PrP106-126 and its two mutants A117V, H111S. Mutated residues are shown in red color, hydrophilic residues in blue and hydrophobic residues in black.

Replica exchange molecular dynamics simulation is an enhanced sampling algorithm that can facilitate the system to escape from local minima in free energy landscape by exchanging temperatures [34]. In REMD method, the replicas of a system are simulated at the same time in different temperatures. After a certain interval, the attempts to swap the temperatures of replicas are made based on the Metropolis Monte Carlo criterion [35]. REMD simulations have been widely used to investigate the structures of peptides with aggregation tendency [36–42]. In this study, AMBER 10 software [43] was used to perform REMD simulations. The conformational spaces of WT, A117V and H111S were investigated by employing the same protocols. The sequences of the three peptides are shown in Fig 1. PrP106–126 consists of a hydrophilic N-terminal region (KTNMKH-) and a long hydrophobic tail (-MAGAAAAGAVVGGLG). The termini of PrP106-126 were capped by acetylating its N-terminal Lys and amidating C-terminal Gly respectively to better simulate the sequence inserted in prion protein. The protonation states of ionizable side chains were specified at pH of 7. Lysine was positively charged and histidine was singly-protonated at the epsilon position in this study. The peptides were modeled using AMBER ff99SB force field [44–46] with the modified Generalized Born solvent model [47]. Compared to AMBER ff99 force field, which has biased tendency towards α-helix structure [48], AMBER ff99SB force field achieves a better balance of prevalent secondary structures such as extended/β-strand and α-helix[44]. Jiang et al. employed AMBER ff99SB force field in combination with the generalized Born/surface area (GBSA) implicit solvent model and successfully predicted the folding pathway of RfaH-CTD [49]. In addition, AMBER ff99SB force field often has been used to study the folding of aggregation-prone peptides [39,50–53]. To define the temperature distributions of the replicas, a web server (http://folding.bmc.uu.se/remd/) was employed [54] and 16 replicas in a temperature range 270–600 K were set.

The initial structures of the three systems were fully extended. Firstly, energy minimization was performed with 2500 steps of steepest decent method followed by 2500 steps of conjugate gradient method to eliminate unnatural collision. Then, the conventional molecular dynamics simulations were carried out for 5 ns to equilibrate each replica at its target temperature. Finally, REMD simulations were performed for 200 ns with an exchange interval of 2 ps. SHAKE [55] algorithm was employed to constrain the bond involved in hydrogen atoms and the time step was 2 fs. The overall exchange rate among replicas was ~40%. The data were collected every 1000 steps (every 2 ps), 100,000 frames were collected in total.

The trajectories of 301.98 K were selected to be analyzed. All the analyses of the three sets of replica exchange molecular dynamics simulations took no account of the first 40 ns and 80,000 frames were used for analyses. Amber [43] and VMD [56] programs were used to perform analyses. The convergence of REMD simulations was assessed by calculating the distributions of different secondary structures and end-to-end distance in two time intervals 40–120 and 120–200 ns. The secondary structures were analyzed using STRIDE [57] algorithm. The end-to-end distance was the distance between the Cα atoms of the 106th residue and the 126th residue. Contacts were considered to be formed when the distance between mass centers of the side chains of two residues was smaller than 6.5 Å. A hydrogen bond was defined to be formed by a donor-acceptor distance not more than 3.0 Å and the angle formed by the donor, hydrogen, and acceptor less than 30°.

## Results and Discussion

### The convergence of REMD simulations

**Fig 2:**
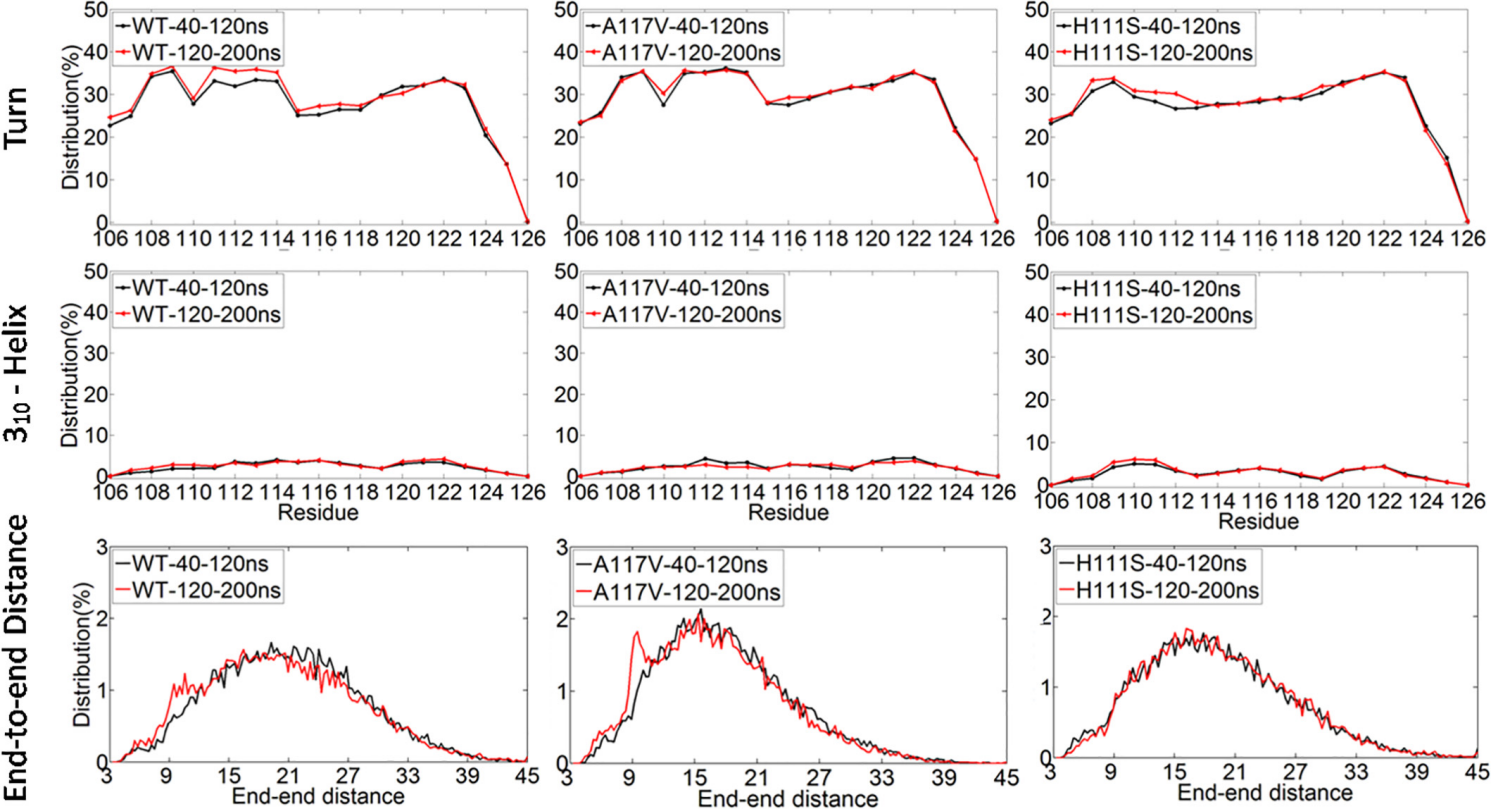
The convergence of replica exchange molecular dynamics simulations at 301.98K for the WT (left) PrP106-126 and its two mutants A117V (middle) and H111S (right).

The convergence of REMD trajectories was assessed by using three metrics and two time intervals (40-120ns, 120-200ns) for the replica at 301.98 K. The three metrics included the distributions of turn contents of residues, the distributions of 3_10_-helix contents of residues and the distributions of end-to-end distances. As shown in Fig 2, the contents of turn as a function of residue index are almost the same in the two time intervals for the WT PrP106-126 and its two mutants A117V and H111S, which indicate that three REMD simulations sample similar conformational spaces in the two intervals. Similarly, the curves of the distributions of 3_10_-helix along the amino acid sequences overlap very well. Furthermore, the distributions of end-to-end distances of three systems in the two time intervals are roughly the same. These results demonstrated that the three REMD simulations reasonably converged in 200 ns.

### The changes of secondary structure

**Fig 3:**
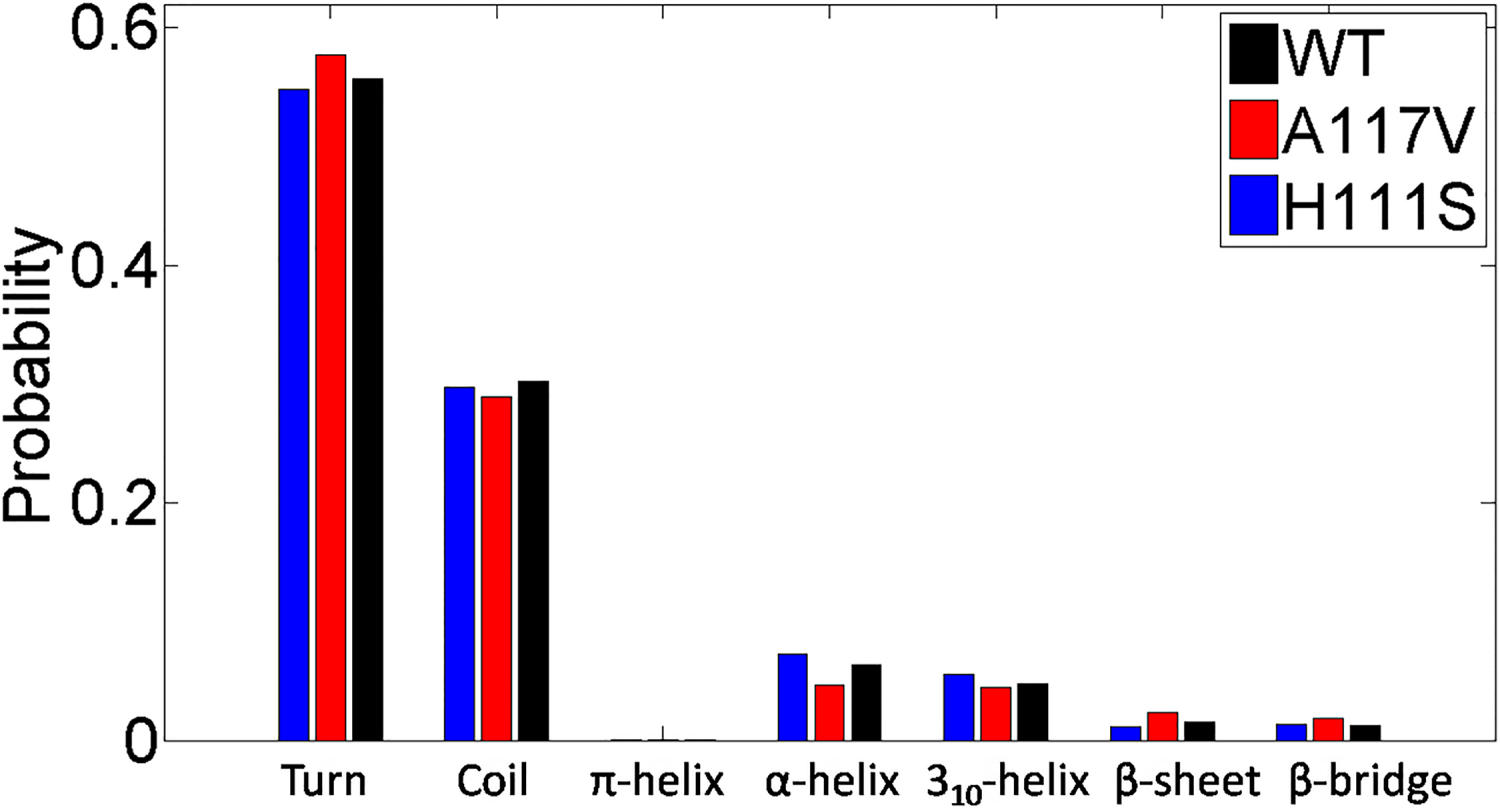
The calculated secondary structure probabilities for the WT PrP106-126 and its two mutants A117V, H111S.

To study how the two mutations affect the conformational properties of PrP106-126, the secondary structure distributions of the three peptides were calculated. The mean values of probability of every secondary structure occurrence for each peptide are shown in Fig 3. The turn and coil dominate with the contents of 55.7% and 30.2% in WT, 57.8% and 29.0% in A117V, and 54.9% and 30.0% in H111S, respectively. Relatively, the helix and β-strand structures remain marginal in the three species. Similarly, experimental study has suggested that PrP106-126 in the acetylated and amide form at its N- and C-termini mainly adopt random coil conformations [19]. Furthermore, Levy et al. observed the spontaneous helix-coil transition of PrP106-126 and suggested that the peptide displayed random coil structure in neutral water solution [21]. However, Bowers et al. have suggested that monomeric PrP106-126 presents high content of β-hairpin structures[24]. The difference may primarily come from the different treatment of termini and the charged termini were proved to enhanced the aggregation of the peptide[33]. In contrast with WT, the secondary structures of α-helix and 3_10_-helix increase upon H111S mutation. Compared to the WT PrP106-126, 13% increase of α-helix and 16% increase of 3_10_-helix are observed in H111S. While in A117V, the propensities to acquire β-sheet and β-bridge structure are enhanced. There are 47% increase of β-sheet content and 45% increase of β-bridge content upon the A117V mutation. The change of secondary structure features may be a possible reason to change the aggregation behavior of PrP106-126. Although A117V and H111S mutants have increased probability to acquire β-sheet and helix respectively compared to the WT PrP106-126, the three species are all intrinsically disordered peptides.

**Fig 4:**
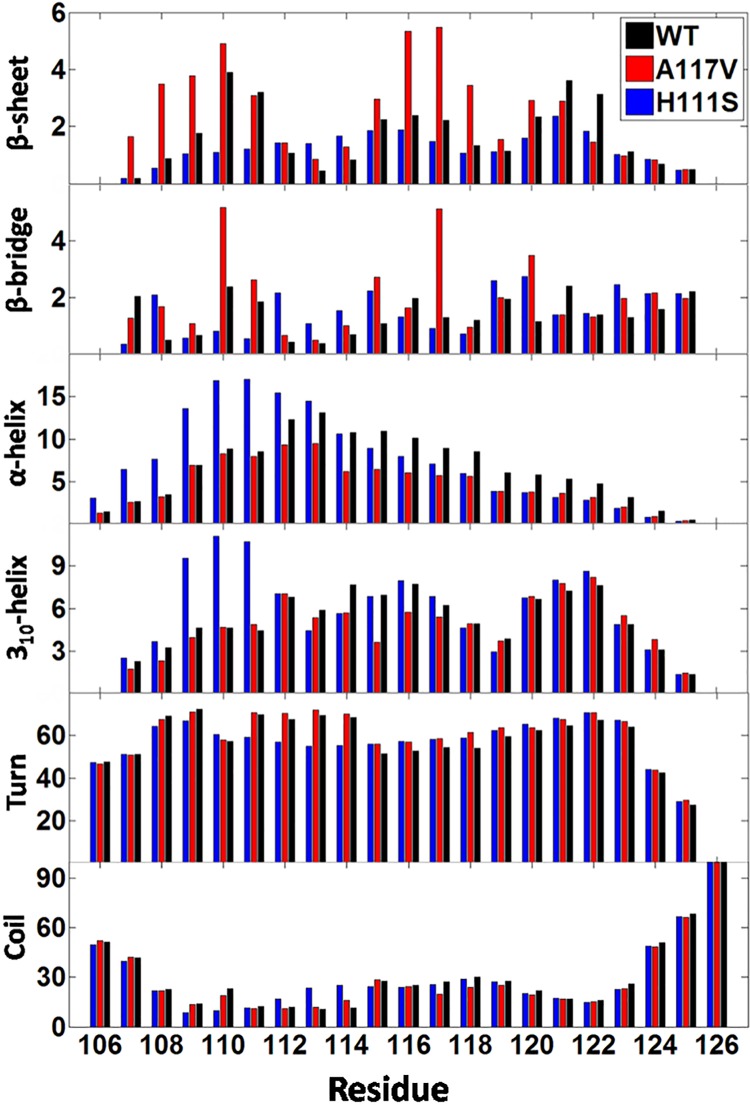
The secondary structure propensities of the residues in the studied peptides. The results were obtained using the time intervals of 40–200 ns.

To further quantify the effects of A117V and H111S mutations on the secondary structures of PrP106-126, we calculated the secondary structure probabilities as functions of the amino acid residues. As shown in Fig 4, the average distributions of turn and coil over each amino acid are similar in the WT PrP106-126 and its two mutants A117V, H111S. When the contents of α-helix and 3_10_-helix over the residues in the three species are compared, a significant trend can be observed. The H111S mutated PrP106-126 shows the greatest propensity to acquire helical structure. Compared to the WT, the H111S mutation enhances the population of helix structure in the N-terminus, especially at residues Met109, Lys110 and Ser111. Relatively, the contents of helical structures in A117V experience a decrease. Among the three peptides, β-sheet propensities are obviously different. The H111S mutant shows a reduced β-sheet propensity when compared to the WT PrP106-126 and the A117V mutant. Upon the A117V mutation, many residues have a higher propensity to form β-sheet structure, such as the residues 107–110 and 116–118. Furthermore, compared to the WT PrP106-126, the A117V mutant is also more inclined to sample β-bridge structure which is considered to be prelude to the formation of β-sheet, especially for the residues Lys110 and Val117. On the contrary, the H111S mutation displays weaker tendency to acquire β-bridge structure. On the whole, the A117V and H111S mutations induce the significant changes in secondary structures of PrP106-126. The A117V mutant has an increased probability to acquire β-structure while the H111S mutation can increase the probability to form helical structure.

Our extensive REMD simulations show that the monomeric A117V mutated PrP106-126 exhibits the higher contents of β-hairpin in contrast with its wild type, consistent with the results of Monte Carlo simulations [22]. The CD spectroscopy experiments suggested that the A117V mutation increased β-sheet structure[30]. Based on the helix propensity scale proposed by Pace and Scholtz[58], serine occurs more frequently in helices than histidine. Therefore, it can be deduced that the H111S mutation may enhance the helix propensity of PrP106-126. Previous experiment also shed light on the effects of H111S on the secondary structures of PrP106-126[33]. It has been suggested that the H111S mutation does not alter the secondary structure of the peptide. This inconsistency also can be attributed to different treatments of terminal amino acids. In the experiments, the H111S mutated peptide was not capped, while the terminal amino acids were both blocked in our study. Furthermore, it has been observed that the terminal blocked PrP106-126 is more helical in this experiment. Herein, it is reasonable that the blocked H111S mutated PrP106-126 may present enhanced tendency to adopt helical configurations than its uncapped analogue.

### Free energy surfaces of the WT PrP106-126 and its two mutants A117V and H111S

**Fig 5:**
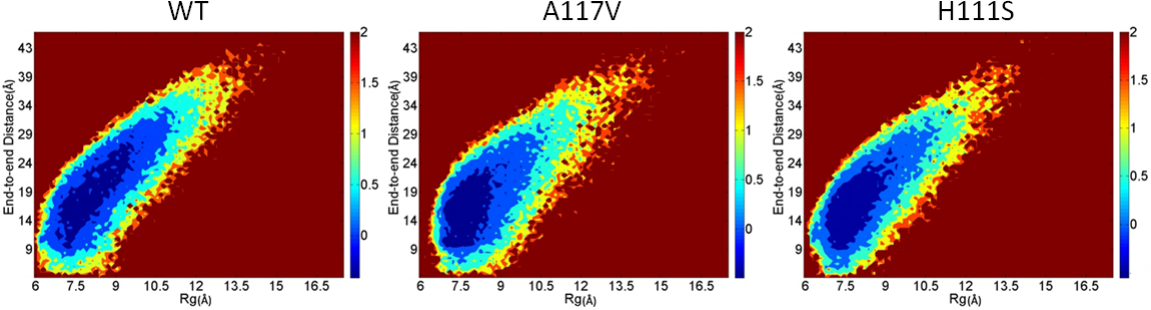
Free energy surface (in Kcal/mol) for WT PrP106-126 and its two mutants A117V, H111S.

To obtain an overall view of conformational distributions of the WT PrP106-126 and its two mutants A117V and H111S, two-dimensional free energy landscapes of the three species were constructed using Rg and end-to-end distance as reaction coordinates. As Fig 5 shows, for each system, the global energy landscape is similar to each other. Multiple local minima with comparable energy are identified and the barriers among the local minima are relatively low, indicating that the conformational conversion can occur easily among different structures. These findings indicate that the three species fail to acquire a dominant structure and all of them display considerable structural diversity. Compared to the WT, local minima of the A117V mutant correspond to the smaller values of Rg and end-to-end distance, which indicates that the inclusion of valine enhances the compactness of the peptide. Similarly, the distributions of the local basins of H111S also suggest relatively compact conformations.

### The comparison of conformational spaces of the WT PrP106-126 and its two mutants A117V, H111S

**Fig 6:**
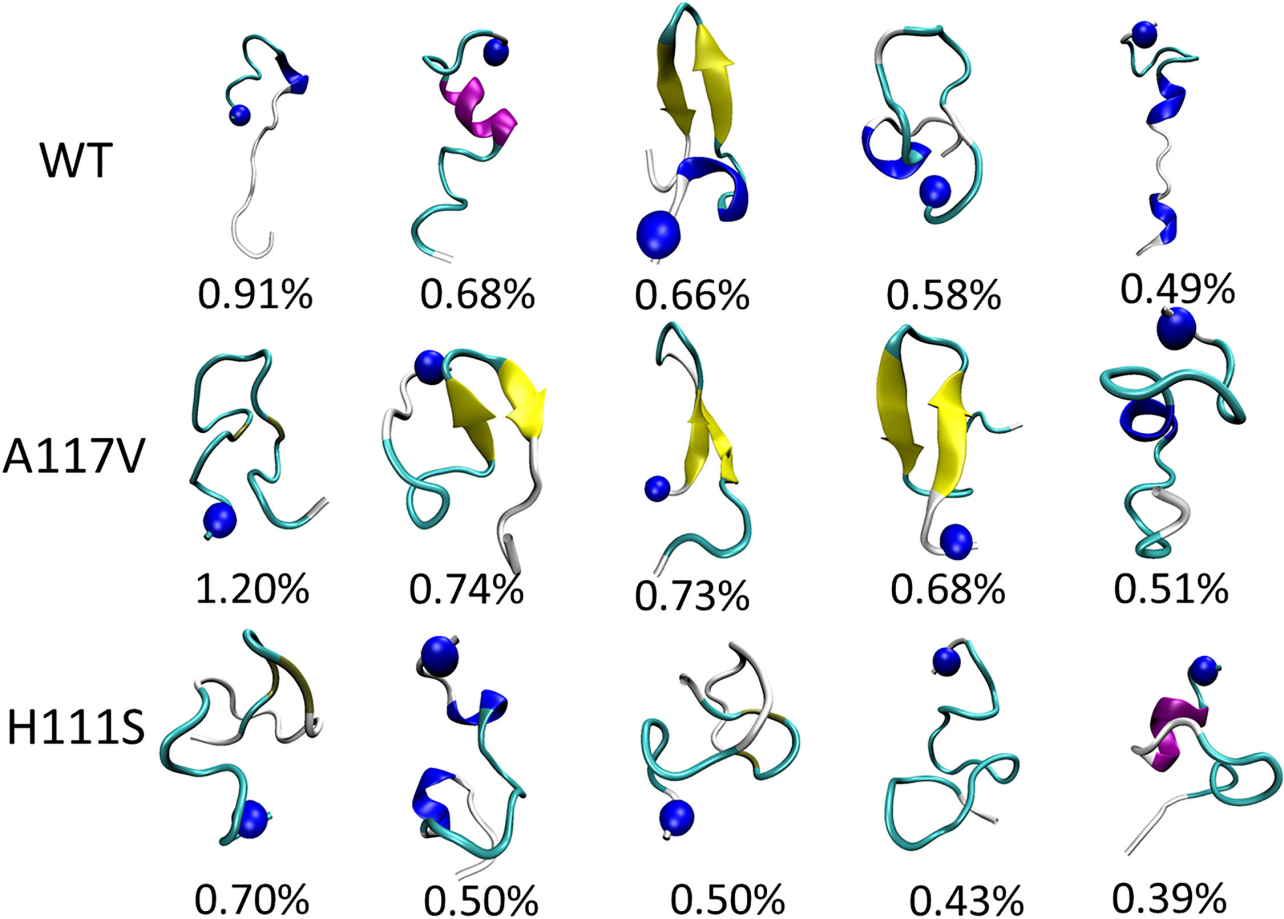
Top five conformations of the WT PrP106-126 and its two mutants A117V, H111S collected by a cluster analysis. The secondary structure was generated using the STRIDE program implemented in VMD. Color scheme for secondary structure: purple for α-helix, yellow for extended β-strand, cyan for turn, and white for random. The N-terminus of each conformation was shown as a blue bead.

Cluster analysis provides a helpful tool to classify the conformational ensemble into clusters with similar geometry. K-cluster algorithm built in MMTSB toolset [59] was employed to cluster the conformations sampled in REMD simulations and the k-means clustering method is widely used to study the conformational distributions of peptides[60–64]. The RMSD cutoff was 4.0 Å and the structures sampled in 40–200 ns were used. A total of 2185, 2190 and 2224 clusters were obtained for WT, A117V, H111S, respectively. The central structures of top five most populated clusters for three species are shown in Fig 6. The populations for the top clusters are very low. The low populations of top clusters could be attributed to the two reasons: firstly, the PrP106-126 and its mutants are intrinsic disordered peptides and the peptides explore large conformational space. The analysis of free energy landscapes and secondary structures also support the viewpoint. There are no local minima in the free energy landscapes of PrP106-126 and its two mutants, which mean that there is no dominating conformations existing and the peptides display significant structural diversities. The main secondary structures of the three peptides are coil and turn, which indicate that the three peptides are very flexible and do not display dominating well-defined secondary structures such as helix and β-sheet. Secondly, we applied REMD simulations, which is an enhanced sampling method. REMD simulations aim to search the conformational space of proteins more efficiently and can sample almost all the possible states of proteins. The variety of sampled configurations in conformational space is enlarged thus it is reasonable that the number of clusters is large and populations of clusters are low; Therefore, the low populations of top clusters do express the real situations and the central structures of the top clusters are representative.

**Fig 7:**
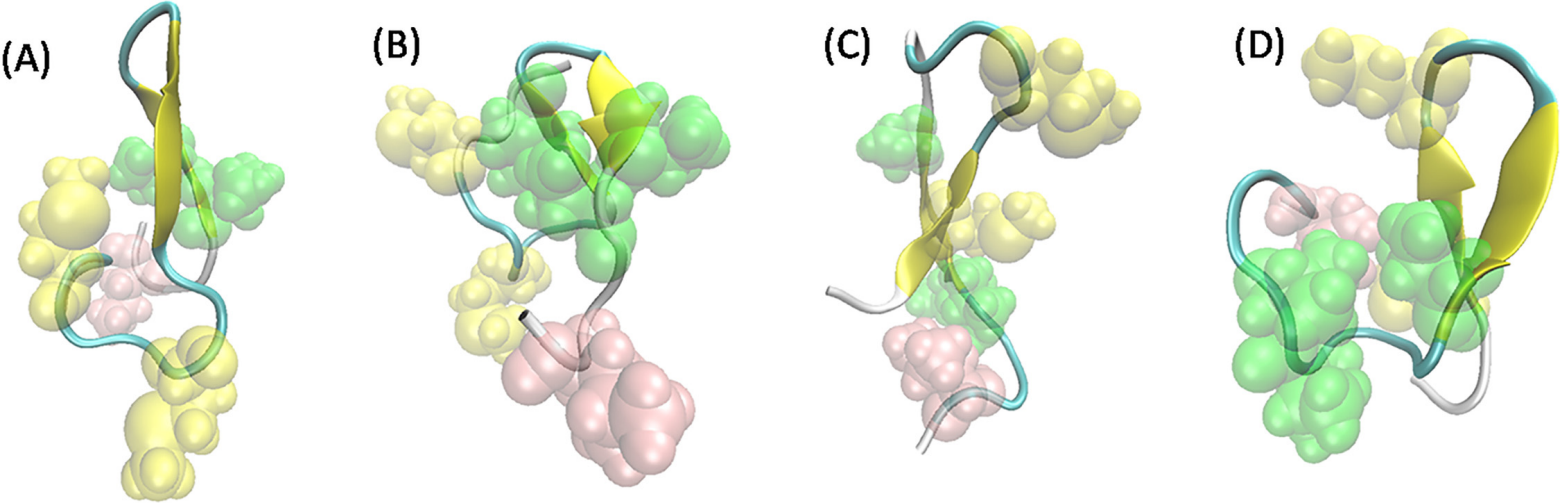
The representative β-hairpin structures sampled by WT and A117V mutated PrP106-126. (A) The most populated β-hairpin structure for the WT, corresponding to the center of its third cluster. (B), (C), (D) Typical β-hairpin structures for A117V mutated PrP106-126, corresponding to its second, third and fourth cluster. The sidechains of hydrophobic residues were shown as spheres and Val, Met, Leu were colored in green, yellow and pink respectively.

Apparently, three species are all flexible and there is no cluster which can dominate the conformational ensemble of three peptides as shown in Fig 6. It can be seen that five representative WT PrP106-126 structures are various with small populations. One of five central conformations displays β-hairpin structure in C-terminal. Similar to the WT, A117V mutated PrP106-126 also evolve into different clustered conformations with the largest population no more than 1.2%. However, three β-hairpin structures are observed in the A117V mutant, indicating its larger propensity to acquire β-hairpin than that in the WT. In the case of H111S, the overall plasticity is also considerable, displaying a strong tendency to form turn and random coil structure and obtaining no β-hairpin. Furthermore, regardless of their position, the formed β-hairpins in the WT and A117V mutated PrP106-126 share common structural feature: hydrophobic residues are almost exposed to solvent. As shown in Fig 7A, hydrophobic residues Met109, Met112 and Val122 are solvent accessible in the β-hairpin structure sampled by the WT PrP106-126. In addition, the β-hairpin structures obtained by the A117V all display the hydrophobic residues exposed to aqueous solution. The exposure of hydrophobic residues will bring the individual PrP106-126 peptide together by the non-specific hydrophobic interaction and facilitate the formation of aggregates.

In our previous study, it has been observed that β-hairpin can act as seed for oligomerization [23]. Furthermore, previous researches on other amyloid-prone peptides have also suggested that β-hairpin formation is an essential step in the misfolding process of these peptides. It has been reported that Aβ_42_ populated more antiparallel β-hairpin structure than Aβ_40_ which is less significant in disease development [65]. Huang et al. also suggested that the presence of β-hairpin segments and extended hydrophobic surfaces facilitated the aggregation of hIAPP [52]. In addition, several molecular dynamics simulations also detected that β-hairpin structure played an important role in the assembly of a range of peptides associated with amyloid formation such as Aβ [38,66] and amylin [67,68]. In this study, we find that the A117V mutated PrP106-126 acquires more β-hairpin structures than the WT PrP106-126 and its H111S mutant, which may provide a plausible molecular basis for why the A117V mutant exhibits stronger propensity of aggregation.

### Residue-residue Interactions

In order to analyze the role of residue-residue interaction in stabilizing the structure of WT PrP106-126 and its two mutants A117V and H111S and probe the impacts of A117V and H111S mutations on the intra-chain interaction of the three species, the contact maps were constructed using the probability of residue-residue contacts between all pairs of amino acids in PrP106-126.

**Fig 8:**
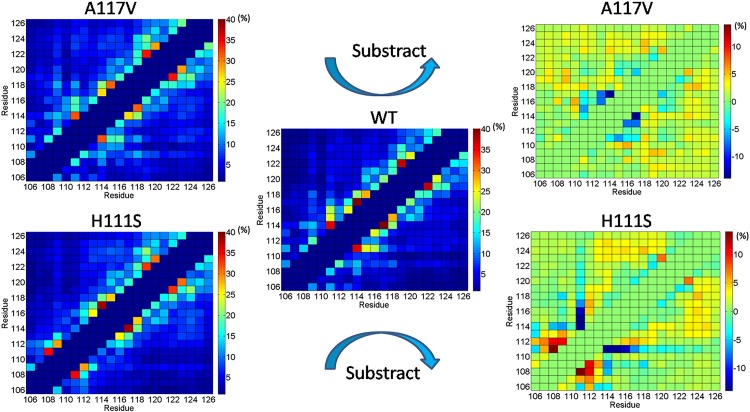
The side chain-side chain contact maps of WT, A117V and H111S in 40-200ns time intervals. The right column represents substracted contact maps between A117V and WT and between H111S and WT. The (i,i), (i,i±1) and (i,i±2) contacts are not included.

To better visually assess the differences in the contact maps upon mutations, we also produced substracted contact maps between A117V and WT and between H111S and WT. As shown in Fig 8, the three species show significant differences in the residue-residue contact propensity. Compared to WT, the inclusion of hydrophobic residue Val117 makes the short-ranged contacts among the residues decrease, especially the interactions between the 114th and the 117th residues. In WT, the contacts between Gly114 and Ala117 represent the strongest interaction among residues with probability of 40%, while the probability of contacts between two residues in the A117V mutant is 26%. In contrast with the impaired short-ranged interactions, the long-ranged interactions are enhanced by the inclusion of the A117V mutation, which indicate that the A117V mutation may increase the conformational freedom of PrP106-126. The enhanced conformational freedom may make the peptide be more inclined to acquire β-sheet structures. Compared to WT, H111S significantly enhances short-range interactions among N-terminal residues of the peptide. The most notable effect is a significant increased contact probability between Asn108 and Ser111. Meanwhile, Met109-Met112 and Asn108-Met112 pair-wise contact probabilities are also relatively higher than that in WT. Compared to histidine, the more hydrophilic serine displays much weaker interactions with the hydrophobic C-terminal residues. The long-range contacts among hydrophobic C-terminal residues are enhanced slightly, which indicated the increased flexibility of the tail of PrP106-126.

**Fig 9:**
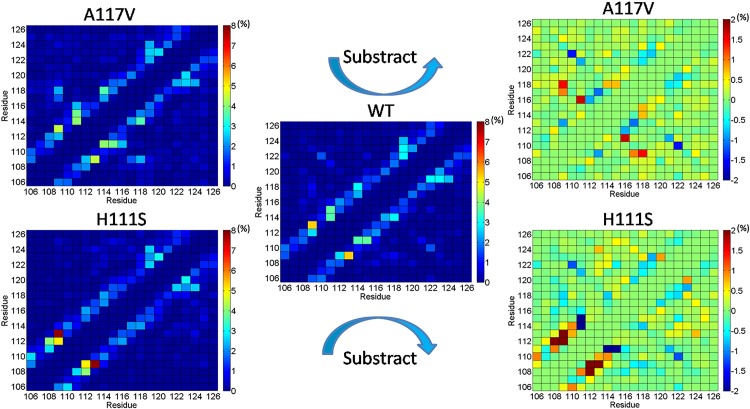
Main chain-main chain hydrogen bond map in 40–200 time intervals. The right column represents substracted hydrogen bond maps between A117V and WT and between H111S and WT.

Hydrogen bonds also play important roles in stabilizing the fold of peptide and in the formation of aggregates. Hence, here the network of main chain-main chain hydrogen bond was analyzed further. For clarity purpose, we also produced substracted hydrogen bond maps between A117V and WT and between H111S and WT. Due to the steric incompatibility, the hydrogen bonds forming between (i,i), (i,i±1), and (i,i±2) residues are rarely observed for the three peptides, resulting in the dark blue diagonal as shown in Fig 9. From Fig 9, the higher probabilities of hydrogen bonds formation of Met109-Met112 and Met109-Ala113 in H111S mutant are observed, which is consistent with its enhanced tendency to acquire α-helix and 3_10_-helix in residues 106–114 compared to the WT and A117V mutated PrP106-126. Compared to the wild type PrP106-126 peptide, residue His111 displays enhanced propensity to form hydrogen bonds with other residues in the A117V mutant. On the contrary, the 111th residue in the H111S mutant displays much weaker tendency to form hydrogen bonds with other residues. As the main driving force stabilizing β-sheet structure, the enhancement of the hydrogen bonds involving His111 in the A117V mutant may increase the content of β-sheet conformations, which may be the critical intermediate in the aggregation of PrP106-126. Our observations indicate the important role of His111 in the folding and aggregation of PrP106-126 on the atomic level. Previous experimental works suggested that His111 is important to the conformational property and aggregation capability of prion protein [69–71]. Salmona et al. have suggested that His111 is central to the conformational changes of PrP106-126 and plays important role in maintaining the β-sheet structures [70]. Wang et al. have also suggested that His111 influences the aggregation behavior of PrP106-126 [71].

By comparing the contact maps and hydrogen bond plots, something of significant interest can be observed. In contrast with the 117th residue in the WT PrP106-126, the residue Val117 in the A117V mutant exhibits weaker tendency to contact with other residues. However, the propensity of this residue to form hydrogen bond is similar to WT. Moreover, the inclusion of Val117 enhances the hydrogen bond formation propensity of its neighbor residues Ala116 and Ala118. The increased frequency of hydrogen bond formation of these residues may indicate that the large hydrophobic side chain of Val117 may be tend to stick out and be solvated, which can spare space for backbone interaction and facilitate hydrogen bond formation. The exposure of hydrophobic Val117 also may increase its propensity to interact with the side chains of the other monomers, which may accelerate the aggregation of PrP106-126. To validate this assumption, the average solvent accessible surface areas (SASA) of the side chain of the 117th residue were calculated in 40–200 ns time interval. The calculated values for A117 in WT PrP106-126 and V117 in A117V mutant are 87 and 137 Å^2^, respectively, indicating that the 117th residue tends to be exposed in A117V mutant. Because hydrophobic interactions play considerable role in the aggregation of PrP106-126, the exposed Val117 may serve as hot spot to drive the aggregation. Daidone et al.[32] also have proposed the notion that the side chain of Val117 in A117V mutated PrP109-122 remains exposed to the solvent. Furthermore, the MD simulations of oligomers performed by this group have suggested that the side chains of Val117 become involved in the formation of hydrophobic cores, which agrees with our assumption that Val117 may be the hot spot in oligomer formation.

**Fig 10:**
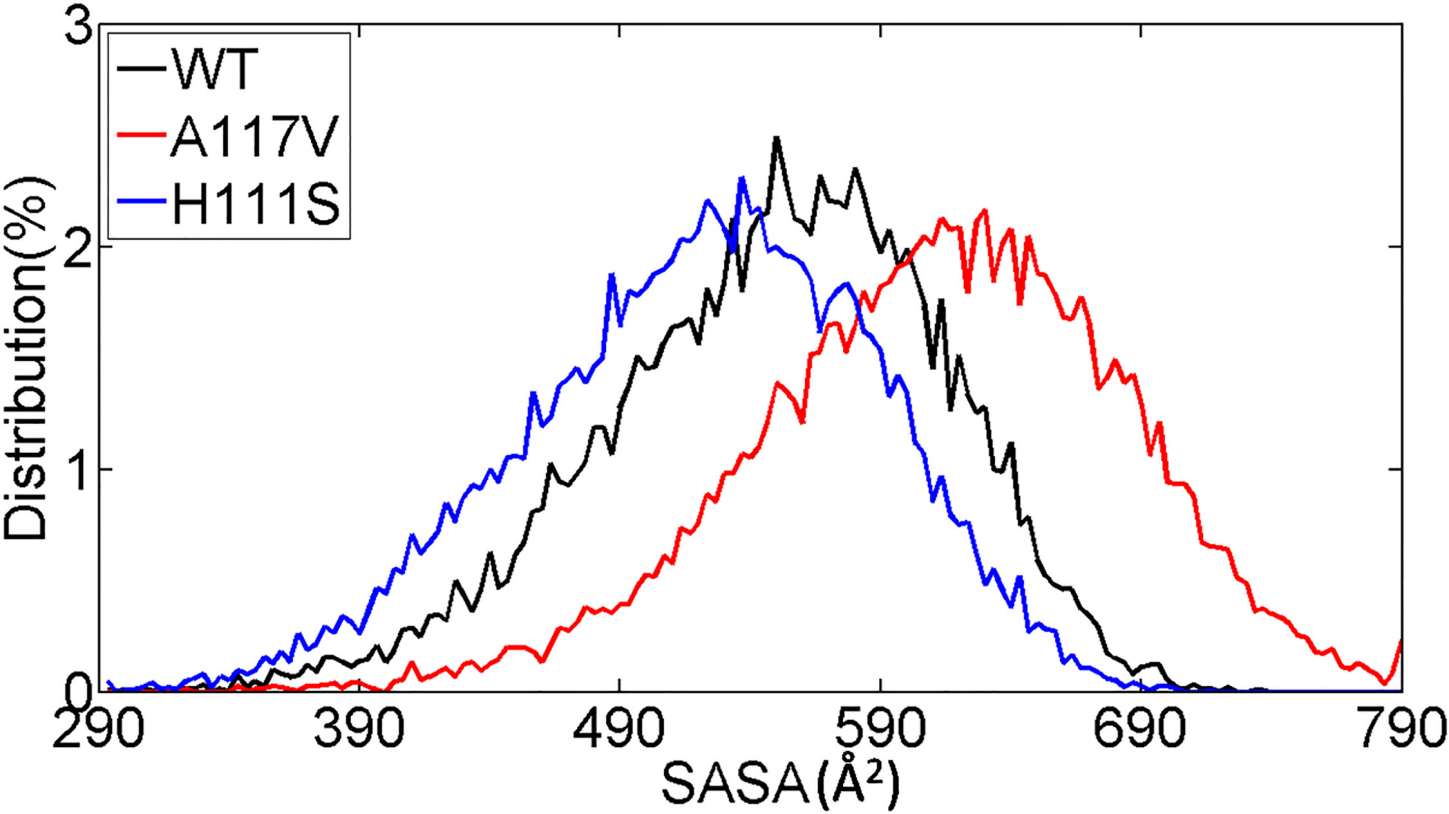
Distributions of hydrophobic solvent accessible areas of WT PrP106-126 and its two mutants A117V, H111S. Hydrophobic SASA of residues Val, Met and Leu were considered.

The overall hydrophobic SASA of the three peptides were also calculated and their distributions were compared. As shown in Fig 10, the A117V mutated PrP106-126 has larger hydrophobic surface areas, with the shift of SASA distributions toward larger values relative to the WT and H111S. Previous computational studies have proved that pathogenic mutations in prion protein such as V210I, T183A, V180I, F198S, T188K/R/A increase the SASA of hydrophobic residues[72,73]. We have also found that the pathogenic A117V mutation induce the hydrophobic residues of PrP106-126 to be more exposed to solvent, which indicate that the effects of these pathogenic mutations on prion protein are similar. Oligomer formation is driven by hydrophobic collapse and a larger exposure of hydrophobic residues in A117V may explain its higher aggregation propensity. In contrast, the H111S mutation induces the decrease in the hydrophobic SASA and the distributions of SASA move to smaller values compared to the WT. The change in hydrophobic SASA in H111S may contribute to its reduced aggregation tendency.

## Conclusions

In this study, the influences of the pathogenic mutation A117V and the protective mutation H111S on the conformational space of PrP106-126 were investigated by REMD simulations. The obtained results show that though the three species are mostly intrinsically disordered, the mutations induce striking morphological differences. The A117V mutated PrP106-126 displays a higher β-hairpin propensity at residues 108–118 than that in WT. The conformational ensemble of H111S also presents significant difference from that of WT PrP106-126. In particular, residues in its N-terminus have higher helix contents. The secondary structural changes may attribute to the alterations of the network of contacts as well as the formation of hydrogen bonds. It is also found that Val117 is less likely to be involved in intra-chain interaction and more prone to be exposed to solvent, rendering itself more favorable for aggregation. Moreover, the overall hydrophobic solvent accessible surface areas of A117V mutant are also increased. The substitution of Ser for His111 makes the N-terminus more inclined to contribute to the intra-chain interaction, which may enhance its propensity to acquire helix structure. Meanwhile, the H111S mutation induces the decrease of hydrophobic solvent accessible surface area. In conclusion, the changes of aggregation behavior in PrP106-126 upon A117V and H111S mutations can be attributed to the two factors. Firstly, the mutations change the structural distributions of PrP106-126 and the different populations of aggregation-prone states result in the distinct rates of aggregation. The A117V mutation may speed up the assembly of PrP106-126 by obtaining more β-hairpin structures. On the contrary, the H111S mutation may inhibit the aggregation of PrP106-126 through acquiring low contents of β-hairpin structure. Secondly, the inclusion of mutations may alter the inter-chain interactions during the aggregation process. The increase of hydrophobic solvent accessible surface areas in A117V may result in the acceleration of fibril formation by enhancing hydrophobic interactions among monomers. Overall, our REMD simulations can deepen our understanding about the misfolding and aggregation mechanism of PrP106-126 and the origin of A117V, H111S mutations affecting the structural features of PrP106-126.
